# Differences between 48 and 72-hour intervals on match load and subsequent recovery: a report from the Brazilian under-20 national football team

**DOI:** 10.3389/fspor.2023.1164454

**Published:** 2024-01-25

**Authors:** Carolina Franco Wilke, Cândido Celso Coimbra, Filipe R. Drummond, Lucas Rios Drummond, Helton Oliveira Campos, Tane Kanope, Guilherme Passos Ramos

**Affiliations:** ^1^ Faculty of Sport, Technology and Health Sciences, St Mary's University Twickenham, London, United Kingdom; ^2^ Department of Physiology and Biophysics, Institute of Biological Sciences, Federal University of Minas Gerais, Belo Horizonte, Brazil; ^3^ Department of Physical Education, Minas Gerais State University, Belo Horizonte, Brazil; ^4^ Department of Biological Science, Minas Gerais State University, Carangola, Brazil; ^5^ Sports Psychology Laboratory, School of Physical Education, Physiotherapy and Occupational Therapy, Federal University of Minas Gerais, Belo Horizonte, Brazil; ^6^ Physiology Department, Aspire Academy for Sports Excellence, Doha, Qatar; ^7^ Brazilian Football Federation, Rio de Janeiro, Brazil

**Keywords:** congested schedule, match interval, youth, soccer, national team, recovery, fatigue

## Abstract

**Purpose:**

To compare the external and internal load and subsequent recovery of football players after international tournament matches separated by 48 h vs. 72 h.

**Methods:**

A total of 14 male football players from the Brazilian National Team, competing in the 2019 South American Under-20 Championship, participated in the study. Match load was quantified using GPS variables and perceived exertion ratings (1). Additionally, before and 13–15 h after each match, players answered questions about the number of hours and quality of sleep, recovery status, and muscle soreness (0–10) and provided a blood sample for creatine kinase and reactive C-protein analysis. Values of all variables were compared between matches played with 48-h intervals (matches 1–4) and 72-h intervals (matches 5–8).

**Results:**

No significant differences in performance or perceptual parameters were observed between matches (*p* = 0.136–0.953). However, CK was higher in matches 1–4 compared to matches 5 and 6; and *Δ*PCR was higher in matches 2 and 3 compared to matches 5 and 6, and in match 4 compared to matches 5 and 8.

**Conclusions:**

After matches with a 48-h rest interval, players showed increased markers of inflammation and muscle damage compared to matches with a 72-h rest interval.

## Introduction

Congested schedule in football is defined as a sequence of two or more matches separated by ≤96 h (1), which is frequently experienced by elite players involved in concomitant tournaments for their clubs and respective national teams throughout a season. Such long and congested calendars also add pressure on health and performance staff, players are exposed to higher levels of fatigue and lower levels of recovery, possibly affecting players' readiness to perform and risk of injury (2, 3). In fact, studies of post-match recovery time indicate that players' strength, sprint, and countermovement jump performance are reduced, as well creatine kinase (CK), reactive C-protein (PCR) concentrations and perceptions of fatigue can be increased after 72 h or more (1, 4, 5). Despite such evidence, international tournament regulations allow consecutive matches to be played at least 48 h apart (6). The South American Under-20 (U20) Championship is an example: it is organized in a two-phase, score-based format, with the interval between matches varying between 48 and 72 h, requiring teams to show a solid campaign throughout.

Previous studies have investigated the effect of consecutive matches with short intervals on the physical demands (7–10) in elite football. Muñoz-Castellanos (2022) observed reduced high-intensity decelerations in the third match compared to the first match played within a 7-day period in elite under-14 (U14), U16, and U19 football players, as well as position-specific differences in distance covered at various speeds between matches. In contrast, other studies did not find differences in external load variables in consecutive matches played with a 72-h interval (7, 8). Given the different findings reported in the literature, Julian et al. (1) concluded that both total and high-intensity distances are not impaired, although players may adapt their activity profiles when playing matches with ≤72-h intervals.

The physical demands of football are influenced by the technical, tactical, and contextual elements of the game (11). In this context, players may self-regulate their activity profile, supported by psycho-physiological aspects, to cope with the overall demands of the match. For example, Mohr et al. (12) compared three matches played in one week (interval between matches 1–2: 72 h, interval between matches 2–3: 96 h). The authors found increased muscle damage (CK), inflammatory markers (PCR), and perception of muscle soreness 48 h after match 2 compared to match 1, but no differences between matches 3 and 1, despite the fact that the players had covered lower distances at high speed in match 2 compared to matches 1 and 3. This suggests that an accumulated fatigue effect (13) may influence recovery if a match is preceded by 72 h compared to 96 h. In addition, fatigue may impair players' response to dynamic disturbances, and mental fatigue may impair technical and tactical performance (14), potentially affecting overall performance and the risk of injury. In fact, Bengtsson (3) demonstrated an approximately 30% increase in football hamstring injuries in the Europa League with matches separated by less than 4 days compared to games separated by more than 6 days. Although the risk of injury is lower in youth groups compared to professional groups, the risk increases with age (15), placing U-20 players at a potentially high risk of injury when submitted to a congested schedule.

As shown, the literature provides evidence for poorer recovery after matches separated by 72 h compared to ≥96 h, but the possible consequences of playing consecutive games separated by the shortest match interval allowed in international tournaments (48 h) have not yet been investigated. Therefore, this study aimed to analyze the differences between two short match intervals (48 h vs. 72 h) in external and internal match load and subsequent recovery markers in an international tournament. To do so, we used data from the 2019 U20 South American Tournament, in which the Brazilian team played all four matches of the first phase with a 48-h interval and all five matches in the second phase with a 72-h interval.

## Materials and methods

### Subjects

The study comprised data from 14 male football players who competed in the 2019 South American U20 Championship for the Brazilian National Team (178.7 ± 5.9 cm, 74.0 ± 7.7 kg, sum of 7 skinfolds: 46.1 ± 5.1 mm), finishing in the fifth place. Inclusion criteria were: (1) being an outfield player; and (2) having played at least one entire match during each phase of the competition. Of the 22 players on the team, three goalkeepers, one outfield player who did not play a full match, and three players who did not play at least one full game in both the first and second phases of the competition were excluded from the analysis. The study was conducted in accordance with the Declaration of Helsinki, 1995, and was approved by the State Ethics Committee of the Federal University of Minas Gerais (47083721.0.0000.5149).

### Study design

This is an observational case study. Data were collected by the team's staff during the championship and used for scientific purposes with the consent of the Brazilian Football Confederation.

### Training and tournament

All players selected for the team participated in a training camp at the Brazilian National Training Centre in Teresópolis. The first phase of the camp consisted of 4 days of initial assessments and familiarization with the training procedures. On the first day, the players underwent medical and anthropometric assessments, followed by 3 days of light-to-moderate- intensity training sessions in the afternoons. At lunchtime before the training session of the third day, blood samples were collected to determine CK and PCR concentrations, which were later used as reference values. After a 5-day Christmas break, the team trained for 17 days in Brazil before traveling to the tournament's host city.

The 2019 South American U20 Championship took place between 14 January and 10 February 2019 (Rancagua, Chile), in two phases. The first phase consisted of four matches separated by 48 h. Following the classification for the second phase, after a 3-day break, the team played five games with a 72-h rest interval (Table 1). Dates, times, opponents, and final results are presented in Table 2.

**Table 1:** Team schedule during the training period and the tournament.

	Days of the month and activity performed						
Training (Brazil)	Dec—17	18	19	20	21	22–25	
	Testing	Training	Training	Blood sample	Friendly Match	Off	
				Training			
	Dec 26 to Jan 11					Jan—12	
	Training camp					Trip to Chile	
Tournament (Chile)	14	15	16	17	18	19	20
	Training	Training	Training	Training	Training	Match 1 18:10–0 × 0 Colombia	Recovery
	21	22	23	24	25	26	27
	Match 2 20:30–2 × 1 Venezuela	Recovery	Match 3 20:30–0 × 1 Chile	Recovery	Match 4 20:30–1 × 0 Bolivia	Recovery	Training
	28	29	30	31	1	2	3
	Training	Match 5 18:30–0 × 0 Colombia	Recovery	Training	Match 6 22:10–0 × 2 Venezuela	Recovery	Training
	4	5	6	7	8	9	10
	Match 7 18:30–2 × 3 Uruguay	Recovery	Training	Match 8 20:50–0 × 0 Ecuador	Recovery	Training	Match 9 22:10–1 × 0 Argentina

**Table 2:** Tournament dates, times, opponents, and results.

Phase	Date	Time	Opponent	Score^a^
1st	19/01/2019	18:10	Colombia	0 × 0
	21/01/2019	20:30	Venezuela	2 × 1
	23/01/2019	20:30	Chile	0 × 1
	25/01/2019	20:30	Bolivia	1 × 0
2nd	29/01/2019	18:30	Colombia	0 × 0
	01/02/209	22:10	Venezuela	0 × 2
	4/02/2019	18:30	Uruguay	2 × 3
	7/02/2019	20:50	Ecuador	0 × 0
	10/02/2019	22:10	Argentina	1 × 0

^a^ Score is presented as Brazil x opponent.

### Pre-match assessments

On the morning of each match, players responded online to a custom-designed questionnaire: number of hours and quality of sleep (rated from 1: Very, very good to 7: Very, very bad), state of recovery (from 0: rested to 10: completely recovered) (16), and muscle soreness (rated from 0: no soreness to 10: extremely sore). The same questionnaire was used in all training camps with the youth national teams; thus, the players were familiarized with it for a minimum of 6 months.

### Match load

Match load parameters were obtained using global positioning system (GPS) units operating at 10 Hz (Statsport®, Newry, Ireland) with an attached accelerometer. The units were switched on 60 min prior to the start of the match and were fitted to the players' upper backs using adjustable neoprene harnesses. Activity profiles were quantified by total distance, distance covered in high-speed running (>20 km.h^−1^) and sprinting (>24 km.h^−1^), the total number of high-intensity accelerations (n. accel) (>3 m.s^−2^), and decelerations (n. decel) (< −3 m.s^−2^). Approximately 30 min after the match, players rated their perceived exertion (RPE; 0: very light to 10: maximum) (16).

### Post-match assessments

On the day following each match (approximately after 13–15 h), a fingertip blood sample was collected for analysis of PCR (Ichroma, Boditech®, Korea) and CK concentrations (Reflotron, Roche®, Switzerland). Additionally, players reported their level of muscle soreness using a visual analog scale. *Δ*PCR and *Δ*CK were calculated as the post-match PCR and CK values minus their respective reference values.

### Statistical Analyses

The data distribution was verified (Shapiro–Wilk). Most variables were non-normally distributed (pre- and post-match recovery, pre- and post-match soreness, RPE, and *Δ*PCR), and comparisons between matches were made using Kruskal–Wallis followed by Dunn's *post-hoc*. Normally distributed variables (total distance, n. accel, n. decel, dist. > 20 km.h^−1^ and dist. sprint) were analyzed using a one-way ANOVA followed by Tukey's *post-hoc* when applicable. All data are shown as median ± interquartile interval for clarity of presentation.

To acknowledge between-player variability, we also performed a repeated-measures analysis with data from those players who played for 90-min in consecutive matches during both phases of the tournament. Due to player rotation and substitution, only data from 5 players who participated in two consecutive matches in each phase were used. One of the players did not wear the GPS unit in game 2, and thus GPS-related variables were compared with an *n* = 4. Matches 1 and 2 (48-h interval) and matches 5 and 6 from the second phase (72-h interval) were compared using either ANOVA (n. accel, n. decel, dist. > 20 km.h^−1^, and dist. sprint) followed by Tukey's *post hoc* when applicable or Friedman (total distance, pre- and post-match recovery, pre and post-match soreness, RPE, *Δ*CK, and *Δ*PCR), followed by Wilcoxon *post-hoc* when applicable.

Effect sizes were calculated as the partial eta squared (*η*^2^) for Kruskal–Wallis and ANOVA, and Kendall's W for Friedman tests.

## Results

**Figure 1:**
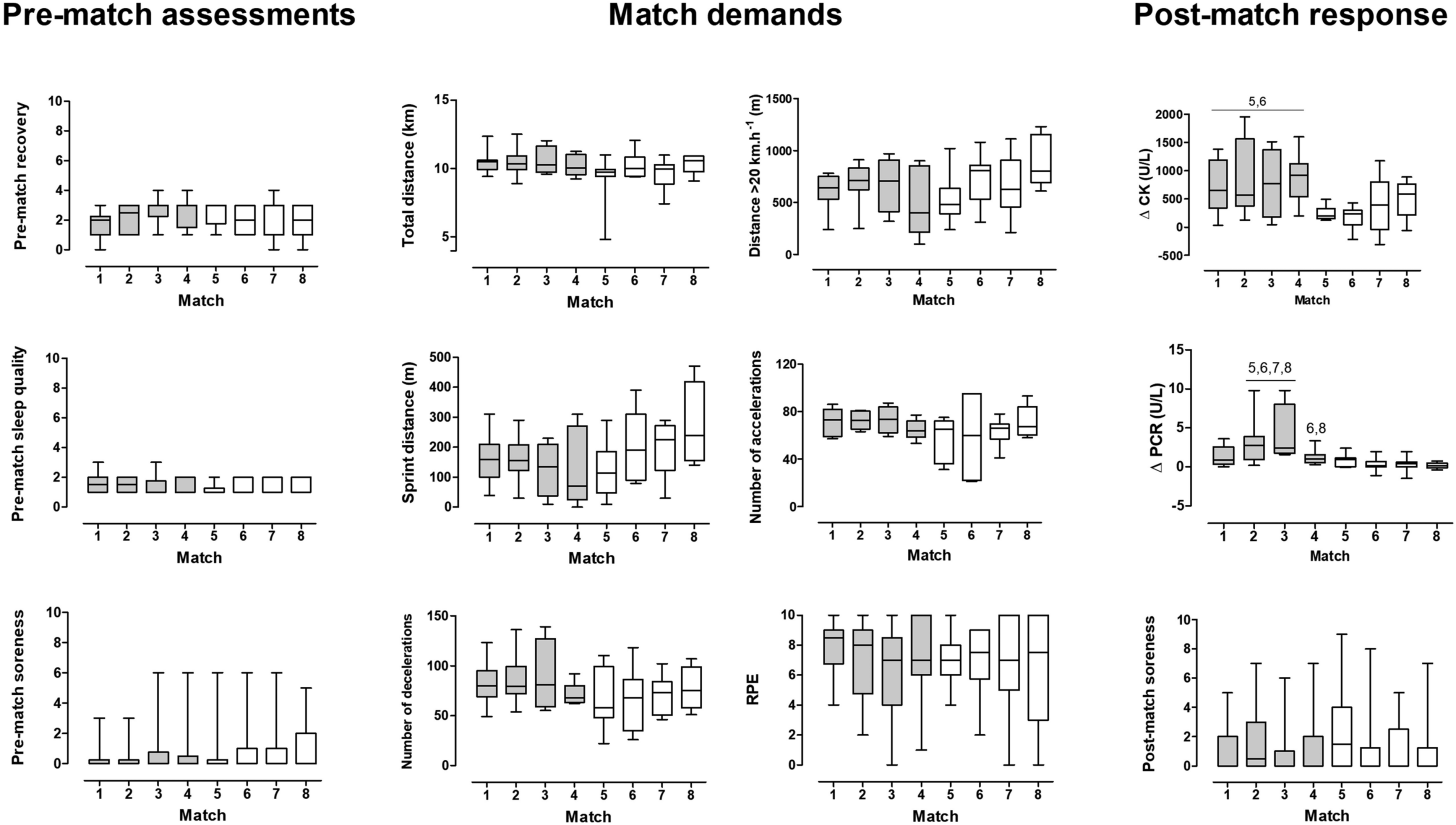
Boxplot of pre-match assessments, match demands, and post-match responses for each match during the 2019 South American U20 championship. Bars in gray represent matches played during the first phase, and bars in white represent matches played during the second phase. ^5^Different from match 5, ^6^Different from match 6, ^7^Different from match 7, ^8^Different from match 8.

There were no differences in pre-match assessments or match demands across the tournament (*p* = 0.092–0.953, *η*^2^ = 0.119–0.227) (Figure 1).

In the post-match assessments, muscle soreness was also similar across matches. However, the Kruskal–Wallis test was significant for *Δ*CK (*p* = 0.005, *η*^2^ = 0.238) and *Δ*PCR (*p* = 0.001; *η*^2^ = 0.330). *Post hoc* analysis showed that *Δ*CK was higher after matches 1 to 4 compared to matches 5 and 6 (*p* < 0.001–0.039). *Δ*PCR was higher in matches 2 and 3 compared to all matches on the second phase, i.e., matches 5–8 (*p* = 0.001–0.045), and *Δ*PCR after match 4 was higher than that in matches 6 (*p* = 0.030) and 8 (*p* = 0.021).

When only data from the same players were included in the analysis, no differences in pre-match assessments were observed between 48-h or 72-h interval situations: soreness (*p* = 0.112; W = 0.400), recovery (*p* = 0.106; *W* = 0.407).

Regarding match load, no differences were observed in distance sprinting (*p* = 0.155, *η*^2^ 0.455), distance traveled above 20 km/h (*p* = 0.590, *η*^2^ = 0.566), or RPE (*p* = 0.063, *W* = 0.487) among matches.

Total distance (*W* = 0.686) and number of decelerations (*η*^2^ = 0.835) were lower in match 5 compared to matches 1 (*p* = 0.020 and *p* = 0.004, respectively) and 2 (*p* = 0.020 and *p* = 0.003, respectively). The number of accelerations (*η*^2^ = 0.714) was lower in match 5 compared to match 2 (*p* = 0.019) and higher in match 6 compared to match 5 (*p* = 0.016).

No differences were observed in post-game soreness (*p* = 0.096, *W* = 0.423). *Δ*CK (*W* = 0.592) and *Δ*PCR (*W* = 0.587) values were higher after match 2 compared to matches 5 (*p* = 0.043) and 6 (*p* = 0.043).

## Discussion

This study showed that players experienced higher post-match markers of inflammation and muscle damage following a 48-h interval between matches compared to 72-h intervals in an international tournament, even with similar match loads.

The similar results in physical performance between matches with 48-h and 72-h intervals (i.e., matches 2, 3, and 4 vs. matches 6, 7, and 8) are in line with a previous study that investigated the differences between 48-h and 72-h intervals in treadmill-simulated conditions in semi-professional football players (17). Other studies on congested schedules have also found similar results, although the between-match interval investigated was longer (72–96 h vs. >96 h) (4, 18). The fact that physical performance during football matches results from a combination of players' physical fitness and technical-tactical demands (4), rather than demands for maximal physical performance, may explain this finding.

**Figure 2:**
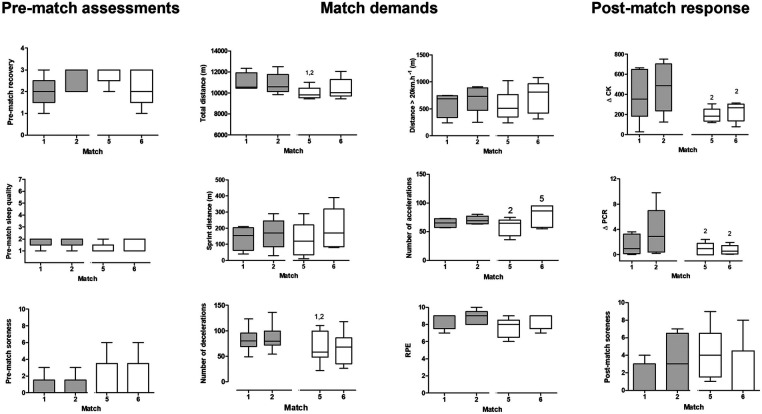
Boxplot of results of pre-, during, and post-game assessments in matches 2 (48 h after match 1) and 6 (72 h after match 5), for players who participated in 90 min of matches 1, 2, 5, and 6. ^1^Different from match 1, ^2^Different from match 2, ^5^Different from match 5.

The physiological stress was higher in a subsequent match when the rest interval was 48 h (match 2) compared to 72 h (match 6). This was assessed by *Δ*CK considering all field players (Figure 1) and by both *Δ*CK and *Δ*PCR considering only repeated measures (players who participated in both matches—Figure 2). Interestingly, these were the only markers that showed the same pattern across players (worse response in match 6 compared to match 2). This was expected based on previous studies showing increased PCR and CK up to 72 h post-match (4). In addition, match load variables that have been demonstrably associated with muscle damage markers, i.e., distance in high-speed running and sprinting (19), were similar between matches separated by 48 h or 72 h (Figure 2). The differences observed in total distance, n. of accelerations, and n. of decelerations may also have influenced the higher CK and PCR concentrations in the first phase compared to the second, although this effect has been shown to be smaller (19, 20). Thus, the 48-h between-match interval appears to lead to a cumulative fatigue effect (21), which has been associated with a higher risk of injury in the long term (18).

Interestingly, the perception of recovery and soreness before the games did not differ between the two intervals, and individual tendencies varied from improved, similar, or worse responses after the 48-h interval compared to the 72-h interval. As no concomitant physiological assessments were available, discussions may be limited. Of note, players were familiarized with the scale and possible interpretations and follow-up decisions by the coaching staff based on their responses (e.g., low recovery scores may indicate poor readiness to play, which may lead to reduced training/game time), whereas playing for the national team is perceived as important for U20 players willing to scale to the professional level. Therefore, we speculate that players may avoid reporting low recovery states immediately before the game. Similar influences have been reported for psychometric measures that rely on player honesty for accuracy (22). Additionally, recovery is a multifactorial phenomenon that may be influenced by factors not assessed here, such as aerobic power, strength, sleep, nutrition, and mood (21, 23).

This study provides valuable insights into its ecological validity in a high-performance setting. However, this is a case study with a limited capacity for extrapolation, and its ecological nature also comes with limitations. First, the fact that players in a national team come from different clubs, being exposed to different training and possibly different stages of the season, brings great variability to their initial assessments. To partially overcome this, the baseline CK concentration was considered to be measured after 4 days of standardized training and routine (time of meals and rest periods). The authors acknowledge the high variability of CK concentration but are supported by studies that also acknowledge its relevance in player fatigue and recovery monitoring programs (13, 20). In addition, the logistics and pressure of an international championship limit the opportunities for data acquisition. Our recovery measures were performed on the day after the match to provide evidence for training load management in the following days, given the short rest intervals between matches. Finally, given the differences in which players were involved in each match, we had limited opportunity for repeated-measures analysis. It is possible that different outcomes would result from a more controlled context, and thus we suggest that further studies be performed to investigate the effects of 48-h match-intervals vs. 72-h intervals or longer.

In conclusion, matches played after a 48-h rest interval were followed by increased markers of inflammation and muscle damage compared to games played after a 72-h interval, with no effect on game demands.

## Data Availability

The raw data supporting the conclusions of this article will be made available by the authors, without undue reservation.
